# Relation between Methylmercury Exposure and Plasma Paraoxonase Activity in Inuit Adults from Nunavik

**DOI:** 10.1289/ehp.1003296

**Published:** 2011-05-04

**Authors:** Pierre Ayotte, Antoine Carrier, Nathalie Ouellet, Véronique Boiteau, Belkacem Abdous, Elhadji Anassour Laouan Sidi, Marie-Ludivine Château-Degat, Éric Dewailly

**Affiliations:** 1 Axe de Recherche en Santé des Populations et Environnementale, Centre de Recherche du Centre Hospitalier Universitaire de Québec, Québec, QC, Canada; 2 Laboratoire de Toxicologie, Institut National de Santé Publique du Québec, Québec, QC, Canada

**Keywords:** Inuit, methylmercury, Nunavik, omega-3 polyunsaturated fatty acids, paraoxonase, PON1, selenium

## Abstract

Background: Methylmercury (MeHg) exposure has been linked to an increased risk of coronary heart disease (CHD). Paraoxonase 1 (PON1), an enzyme located in the high-density–lipoprotein (HDL) fraction of blood lipids, may protect against CHD by metabolizing toxic oxidized lipids associated with low-density liproprotein and HDL. MeHg has been shown to inhibit PON1 activity *in vitro*, but this effect has not been studied in human populations.

Objectives: This study was conducted to determine whether blood mercury levels are linked to decreased plasma PON1 activities in Inuit people who are highly exposed to MeHg through their seafood-based diet.

Methods: We measured plasma PON1 activity using a fluorogenic substrate and blood concentrations of mercury and selenium by inductively coupled plasma mass spectrometry in 896 Inuit adults. Sociodemographic, anthropometric, clinical, dietary, and lifestyle variables as well as *PON1* gene variants (rs705379, rs662, rs854560) were considered as possible confounders or modifiers of the mercury–PON1 relation in multivariate analyses.

Results: In a multiple regression model adjusted for age, HDL cholesterol levels, omega-3 fatty acid content of erythrocyte membranes, and *PON1* variants, blood mercury concentrations were inversely associated with PON1 activities [β-coefficient = –0.063; 95% confidence interval (CI), –0.091 to –0.035; *p* < 0.001], whereas blood selenium concentrations were positively associated with PON1 activities (β-coefficient = 0.067; 95% CI, 0.045–0.088; *p* < 0.001). We found no interaction between blood mercury levels and *PON1* genotypes.

Conclusions: Our results suggest that MeHg exposure exerts an inhibitory effect on PON1 activity, which seems to be offset by selenium intake.

Exposure to methylmercury (MeHg) from fish consumption may be associated with an increased risk of heart diseases, particularly myocardial infarction (Stern 2005). The main evidence supporting this relationship was provided by Finnish studies conducted among non-fatty-fish eaters (Salonen et al. 1995). Other populations consume different species of fish and in some cases marine mammals, and their risk of cardiovascular effects may be related not only to MeHg exposure but also to nutrients that may counteract the toxic effects of MeHg, such as omega-3 polyunsaturated fatty acids (n-3 PUFAs) and selenium, that are high in some marine foods. Inuit populations living in circumpolar countries have had lower incidences of coronary heart disease (CHD), particularly ischemic diseases, than those of more southern populations living in industrialized areas (Bang et al. 1971; Corcoran and Rabinowitch 1937; Kjaergaard et al. 2009). However, changes in some aspects of their diet and lifestyle that have occurred over the last decades have been associated with increased risk of CHD (Château-Degat et al. 2010; Counil et al. 2009; Jorgensen et al. 2008; Young et al. 2007). In particular, a decreased intake of food items that are rich in n-3 PUFAs or selenium (sea mammal fat and skin) could exacerbate the toxic effects of MeHg on the cardiovascular system.

The mechanism of action through which MeHg increases the risk of CHD remains to be identified. Oxidative damage generated by MeHg has been suggested as a possible early biological effect resulting from MeHg exposure, in turn causing lipid peroxidation, inflammation, and endothelial cell damage (Grotto et al. 2009). Animal studies have shown that MeHg can induce oxidative stress directly by catalyzing reactions that produce reactive oxygen species (Grotto et al. 2009; Lin et al. 2006). Alternatively, oxidative stress may indirectly result from the interaction of MeHg with thiol or selenol groups in antioxidant molecules such as glutathione and enzymes such as glutathione peroxidase and thioredoxin reductase (Carvalho et al. 2008; Khan and Wang 2009).

Human paraoxonase (PON1) is an enzyme associated with the high-density lipoprotein (HDL) particle that inhibits oxidation of low-density lipoprotein (LDL) and HDL through hydrolysis of lipid peroxides (Aviram 2000; Mackness et al. 1998). Knockout mice lacking the *PON1* gene develop atherosclerosis more rapidly than do wild-type mice (Shih et al. 2000). Hence, it has been suggested that PON1 inhibits the atherosclerotic process by preventing LDL oxidation in the arterial wall. The involvement of PON1 in the pathogenic sequence is further supported by the fact that decreased PON1 activity is associated with an increased prevalence of atherosclerosis (Jarvik et al. 2000; Mackness et al. 2001) and incidence of cardiovascular disease (Mackness et al. 2003).

Three common single-nucleotide polymorphisms (SNPs) within the *PON1* gene appear to be the strongest determinants of serum PON1 activity (Costa et al. 2005; Ferré et al. 2003). Two SNPs occur in the coding region: the first involves a change of methionine (M allele) for leucine (L allele) at position 55 (L55M; rs854560); the second involves the substitution of arginine (R allele) for glutamine (Q allele) at position 192 (Q192R; rs662). The latter has been shown to significantly modulate the activity of PON1 toward its various substrates. The third SNP, –108C/T (rs705379), is located in the promoter region and has a major influence on *PON1* expression (Brophy et al. 2001).

Despite the predominance of genetic influences, several other factors can modulate serum PON1 activity, such as age, drugs, diseases, smoking, alcohol, diet, and environmental chemicals (Costa et al. 2005). Because of the known capacity of toxic metals to inhibit enzymes, *in vitro* experiments have been conducted to investigate the inhibitory effects of toxic metals on PON1 activity. Gonzalvo et al. (1997) first reported that copper and mercurials were potent inhibitors of PON1 activity in human liver microsomes. Similar results were obtained in other *in vitro* experiments conducted with pooled human serum of subjects with the *PON1*_Q192_ genotype; manganese (Mn^2+^), cobalt (Co^2+^), cadmium (Cd^2+^), and nickel (Ni^2+^) also inhibited PON1 activity in these experiments (Debord et al. 2003). Subsequent studies have reported that metals were more effective inhibitors of the PON1_R192_ isozyme than of the PON1_Q192_ isozyme (Cole et al. 2002; Costa et al. 2005; Gençer and Arslan 2009). Despite these compelling results from *in vitro* experiments, treatment of mice with Cd, MeHg, or dietary iron, leading to metal serum concentrations > 1 μM, did not alter serum or liver PON1 activity (Cole et al. 2002). To our knowledge, the association between MeHg exposure and PON1 activity has yet to be tested in humans and may prove to be of public health relevance in Inuit who are highly exposed to MeHg through fish and marine mammal consumption (Fontaine et al. 2008). This population is also exposed to lead, a metal whose blood levels were significantly associated with decreased serum PON1 activity in lead workers (Li et al. 2006).

We conducted a comprehensive health survey in the Inuit population of Nunavik (Québec, Canada) during the fall of 2004. In the course of this study, we investigated the relation between blood mercury concentrations and plasma PON1 activities in 896 Inuit adults living in Nunavik, while taking into account the potential protective role of selenium, which has been shown to counteract the toxicity of mercurials (Khan and Wang 2009). We also investigated the possible confounding or modifying role of several factors, including *PON1* gene variants.

## Materials and Methods

*Study population and data collection.* This health survey was conducted in Nunavik, a northern region of Québec where approximately 9,500 Inuit live in 14 communities along the coasts of Hudson Bay, Hudson Strait, and Ungava Bay. Informed consent was obtained from all participants before enrolling them in the study, which was approved by the Comité d’éthique de la recherche de l’Université Laval and the Comité d’éthique de santé publique du Québec. The target population of this study was permanent Inuit residents of Nunavik from 18 to 74 years of age. To obtain a standard representation of the target population, the study used a stratified random sampling of private Inuit households, with the community being the stratification variable.

Several self-administered and interviewer-completed questionnaires were used to obtain information regarding demographics, lifestyle habits, nutrition, and health indicators. In addition, individuals were asked to participate in a clinical session where blood samples were taken and anthropometric measurements were performed. Data on body composition (body fat percentage, lean body mass) were obtained by bioelectrical impedance using the Tanita TBF-300A analyzer (Tanita Corporation of America, Inc., Arlington Heights, IL, USA). Medical history and related drug consumption of each participant were documented by medical chart review.

Among the 677 contacted households, 521 agreed to participate (household response rate of 77.8%), 1,056 individuals signed a consent form, and 917 people agreed to the collection of blood samples for clinical and toxicological analyses (final participation rate of 67%). Because of various problems (insufficient plasma volume, broken vials), we measured PON1 activity for 899 individuals. Three participants were excluded from the database because of extreme values of blood mercury (> 800 nmol/L) and blood selenium (> 30 μmol/L).

Data on the consumption of traditional foods were obtained from a food frequency questionnaire, which was designed to measure season-specific consumption of food items derived from fishing and hunting during the year before the survey. Season-specific intakes were computed by multiplying the daily consumption frequency by the portion size for each food item and season. Season-specific intakes were then averaged over the four seasons to yield the mean annual consumption (grams per day). The following three composite variables were created: *a*) wild fish comprised clam, mussel, oyster, scallop, seaweed, urchin, arctic char, cod, whitefish, trout, salmon, dried fish, and other fish such as pike, cisco, and walleye; *b*) marine mammal fat comprised beluga and seal fat; and *c*) marine mammal meat comprised beluga meat, dried beluga, beluga muktuk (skin), beluga liver, other parts of beluga, seal meat, walrus meat, igunak (aged meat), seal kidney, seal liver, other parts of seal, and walrus parts.

*Plasma PON1 activity.* Blood was collected from a cubital vein in a 6-mL plastic Vacutainer containing sodium heparin as the anticoagulant (BD Medical, Mississauga, ON, Canada). Blood was centrifuged within 2 hr of collection, and the plasma was stored frozen at –80°C until time of analysis. Although in most previous studies PON1 activity has been measured in serum samples, the suitability of heparin- plasma samples was demonstrated by Ferré et al. (2005). PON1 activity was measured in 5 μL plasma, diluted to a final concentration of 2%, using the EnzChek Paraoxonase Assay Kit (Molecular Probes, Invitrogen, Carlsbad, CA, USA). This highly sensitive, homogeneous fluorometric assay for the organophosphatase activity of PON1 is based on the hydrolysis of a fluorogenic organophosphate analog (7-diethylphospho-6,8-difluor-methylumbelliferyl). The intensity of emitted light was measured with a Victor2 plate reader (PerkinElmer–Cetus Life Sciences, Boston, MA, USA) using 355- and 460-nm filters for excitation and emission, respectively. Enzymatic activity was calculated by converting fluorescence intensity into activity using a standard curve. Activity was expressed as kilounits per liter, where one unit of PON1 is defined as the amount of enzyme that produces 1 nmol fluorescent product per minute at 37°C. Aliquots of a plasma sample obtained from a volunteer were stored frozen at –80°C along with the study samples; this “control” sample was analyzed in each analytical batch. The mean value was 13.3 kU/L, and the between-day coefficient of variation was 7.6% (*n* = 23).

*Metal analyses.* Mercury, selenium, and lead concentrations were determined in whole-blood samples collected from a cubital vein in a 6-mL plastic Vacutainer containing potassium EDTA as the anticoagulant (BD Medical). Blood samples were stored frozen at –80°C until time of analysis. Determination was performed by inductively coupled plasma mass spectrometry on an ELAN DRC II instrument for mercury and selenium and an Elan 6000 instrument for lead (Perkin-Elmer SCIEX, Concord, ON, Canada). Blood samples were diluted 20-fold in an ammonia solution before analysis. Limits of detection for mercury, lead, and selenium were 0.5 nmol/L, 1 nmol/L and 0.1 μmol/L, respectively, and each run of samples included a standard. Between-day coefficients of variation for mercury, lead, and selenium measurements were 2.1%, 2.8%, and 6.1%, respectively. Analyses were performed by the Laboratoire de Toxicologie of the Institut National de Santé Publique du Québec, which has ISO 17025 accreditation and participates in the quality assurance/quality control programs of the Canadian Northern Contaminants Program and the Arctic Monitoring Assessment Program.

*Plasma lipids.* Concentrations of total cholesterol and triacylglycerol in plasma samples were determined by enzymatic methods using a Hitachi 917 autoanalyzer and reagents, both from Roche Diagnostics (Laval, QC, Canada). HDL cholesterol (HDL-C) was measured directly by selectively inhibiting reaction with other lipoproteins. LDL cholesterol (LDL-C) was calculated using the Friedewald formula [LDL-C (grams per liter) = total cholesterol (grams per liter) – HDL-C (grams per liter) – triacylglycerol (grams per liter)/5] (Tremblay et al. 2004). Lipid analyses were performed at the Centre de Recherche sur les Maladies Lipidiques (Centre de Recherche du Centre Hospitalier Universitaire de Québec, Quebec, QC, Canada).

*Fatty acid analysis in erythrocyte membranes.* The fatty acid composition of phospholipids in erythrocyte membranes was also determined at the Centre de Recherche sur les Maladies Lipidiques. Briefly, total lipids were extracted with a chloroform/methanol mixture, and the phospholipid fraction was isolated by thin-layer chromatography (Shaikh and Downar 1981). Fatty acids were transmethylated (Lepage and Roy 1988), and the resulting fatty acid methyl esters were analyzed by capillary-column gas chromatography on a Hewlett-Packard 5890 series II gas chromatograph (Hewlett-Packard, Mississauga, ON, Canada) equipped with a fused silica column (DB-23; 30 m × 0.25 mm inner diameter × 0.25 μm thickness; Agilent Technologies, Mississauga, ON, Canada) and a flame ionization detector. Concentrations of individual fatty acids in membrane phospholipids were expressed as a percentage of total fatty acids (weight basis). The sum of eicosapentaenoic acid (EPA; 20:5n-3) and docosahexaenoic acid (DHA; 22:6n-3) content of erythrocytes was used as a surrogate of marine food consumption. This sum has been referred to as the omega-3 index and has been shown to be inversely associated with risk for CHD mortality (Harris and Von Schacky 2004).

*Genotyping.* A subsample of 659 participants gave their consent to the genotyping measures. Variants of rs662, rs854560, and rs705379 were originally analyzed using Sequenom technology at McGill University and Génome Québec Innovation Centre (Montréal, QC, Canada). One SNP failed (rs705379), and the call rate was > 99.7% for the remaining two SNPs. The error rate of this technology is < 0.5% according to duplicate and control samples tested at the Génome Québec Innovation Centre. The rs705379 SNP was reanalyzed with the TaqMan technology at the same center with success and a call rate of 99.9%. TaqMan technology has an average error rate of 0.1%. All genotypes were in Hardy–Weinberg equilibrium.

*Statistical analyses.* To meet normality assumption, body mass index (BMI), fat mass, blood mercury and lead levels, plasma HDL-C and LDL-C concentrations, EPA content of erythrocyte membranes, PON1 activity, and food consumption rates were log transformed, whereas an inverse transformation was retained for blood selenium levels. Transformations were selected based on both normality test (Kolmogorov–Smirnov) and bivariate relationships between variables. We used the BoxCox macro developed by Michael Friendly to achieve the best transformation (http://www.datavis.ca/sasmac/boxcox.html). Pearson’s correlation coefficients were used to investigate crude relationships between PON1 activity (kilounits per liter) and blood concentrations of mercury (nanomoles per liter), selenium (micromoles per liter) and lead (micromoles per liter), age (years), BMI (kilograms per square meter), fat mass (kilograms), HDL-C (millimoles per liter), LDL-C (millimoles per liter), marine food consumption (grams per day), and n-3 PUFAs (percent). Student’s *t*-tests or analyses of variance were used to test differences in mean PON1 activity according to sex, alcohol consumption (daily, weekly, monthly, never, or yearly), daily cigarette consumption (0, 1–10, 11–24, ≥ 25), genotypic variables (rs705379, rs662, rs854560), statin intake, cardiovascular diseases, diabetes, and dyslipidemia (for disease definitions, see Château-Degat et al. 2010). We subsequently conducted multiple linear regression analyses to examine the relation between blood mercury level and plasma PON1 activity, while controlling for several potential confounding factors. Interactions terms (mercury × *PON1* variants, mercury × selenium, mercury × n-3 PUFAs) were included in initial models. Generalized estimating equations techniques were used to take into account the clustering structure of the data (household was the sampling unit). Continuous explanatory variables were also centered and scaled for multicollinearity and interpretive purposes. Furthermore, because of a considerable number of missing values for gene variants (27% of participants), multiple linear regression analysis was rerun after using multiple imputation methods on genotypic variables. The Kolmogorov–Smirnov test of normality was conducted on regression residuals. Contrasts were used to test a linear combination of mercury quartiles coefficients (trend test). Because of the complex design sampling and to achieve precision in the parameter estimates, statistical tests were weighted and bootstrapped (balanced repeated replication). All analyses were performed using SUDAAN software (version 10.0.1; Research Triangle Institute, Research Triangle Park, NC, USA) and SAS software (version 9.2; SAS Institute Inc., Cary, NC, USA). We selected an α-level of 0.05 for hypothesis testing.

## Results

Table 1 lists selected characteristics of our study group, which is composed of 896 Inuit adults between 18 and 74 years of age; the mean age of the group was 36.6 years. We noted a BMI value exceeding 30 kg/m^2^ in 26.0% of men and 31.1% of women. More than two-thirds of the participants were current smokers, and their average consumption was 15.7 cigarettes/day for men and 12.5 cigarettes/day for women. The vast majority of men (92.8%) and women (84.3%) declared drinking alcohol. More than 90% of men and women declared consuming marine mammal meat and wild fish, whereas about 70% reported eating marine mammal fat. Daily wild fish consumption was more than double that of marine mammal meat and exceeded by an order of magnitude the consumption of marine mammal fat. According to the medical files, about 8% of men and 7% of women had dyslipidemia and were taking hypolipemic drugs (statins) at the time of the study. Cardiovascular diseases were affecting 17% of men and 20% of women, whereas only 3% of men and 4% of women had diabetes.

**Table 1:** Selected characteristics of Inuit adults participating to the Nunavik Health Survey, 2004.

Men			Women		
Characteristic	*n*	Mean (95% CI)	*n*	Mean (95% CI)
Age (years)		405		36.5 (35.9–37.0)		491		36.7 (36.2–37.2)
Weight (kg)		380		74.4 (72.8–76.0)		438		65.8 (64.5–67.1)
Height (cm)		390		166.1 (165.5–166.7)		447		153.9 (153.6–154.4)
BMI (kg/m^2^)		380		26.9 (26.4–27.4)		438		27.7 (27.2–28.2)
Fat mass (kg)		379		16.7 (15.7–17.6)		438		22.3 (21.3–23.2)
EPA (%)		405		1.5 (1.4–1.6)		491		1.7 (1.6–1.8)
DHA (%)		405		4.9 (4.8–5.1)		491		5.7 (5.6–5.8)
HDL-C (mmol/L)		402		1.5 (1.4–1.6)		490		1.8 (1.7–1.9)
LDL-C (mmol/L)		402		2.8 (2.7–2.9)		490		2.7 (2.6–2.8)
Blood lead (μmol/L)		405		0.27 (0.25–0.29)		491		0.21 (0.20–0.23)
Consumption (g/day)								
Marine mammal fat		346		4.4 (3.4–5.4)		401		4.9 (3.7–6.1)
Marine mammal meat		347		25.9 (21.2–30.5)		404		18.5 (15.4–21.6)
Wild fish		347		59.8 (52.2–67.4)		404		51.1 (44.5–57.7)
No. of cigarettes/day (%)								
0		100		27.2 (23.4–31.4)		96		21.6 (18.5–25.2)
1–10		83		24.5 (20.4–29.0)		174		40.6 (36.2–45.1)
11–24		113		32.4 (27.8–37.3)		122		27.8 (24.0–32.0)
≥ 25		58		15.9 (12.7–19.8)		42		10.0 (7.8–12.8)
Frequency of alcohol consumption (%)								
Daily		26		7.5 (4.4–10.2)		34		8.3 (6.0–11.3)
Weekly		83		23.1 (19.3–27.3)		74		18.7 (15.5–22.3)
Monthly		82		24.0 (19.8–28.7)		95		21.9 (18.6–25.6)
Never or yearly		161		45.5 (40.6–50.4)		227		51.2 (47.2–55.2)
Statin intake (%)								
Yes		34		7.8 (5.9–10.2)		36		7.1 (5.3–9.4)
No		371		92.2 (89.8–94.1)		455		92.9 (90.6–94.7)
Cardiovascular disease*a* (%)								
Yes		70		16.6 (13.7–19.9)		94		19.7 (16.7–23.0)
No		335		83.4 (80.1–86.3)		397		80.3 (77.0–83.3)
Diabetes (%)								
Yes		12		3.0 (1.8–4.9)		20		3.88 (2.6–5.7)
No		393		97.0 (95.2–98.2)		455		96.1 (94.3–97.4)
Dyslipidemia (%)								
Yes		35		8.0 (6.1–10.4)		36		7.1 (5.3–9.4)
No		370		92.0 (89.6–93.9)		455		92.9 (90.7–94.7)
CI, confidence interval. **a**Includes all cardiovascular diseases except hypertension.								

**Figure 1:**
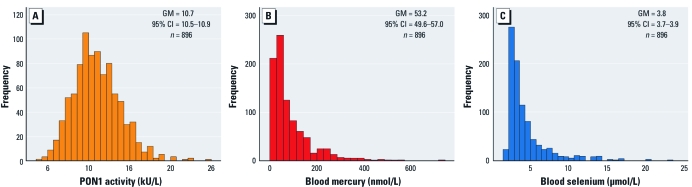
Frequency distributions of PON1 activity (*A*), blood mercury concentrations (*B*), and blood selenium concentrations (*C*) in Inuit adults from Nunavik, 2004. Abbreviations: CI, confidence interval; GM, geometric mean.

Figure 1A presents the frequency distribution of plasma PON1 activity. The geometric mean was 10.7 kU/L, with values ranging from 4.3 to 24.6 kU/L. Frequency distributions of blood mercury concentrations (Figure 1B) and blood selenium concentrations (Figure 1C) were markedly skewed to the right. Geometric mean values were 53.2 nmol/L (range, 0.4–720) and 3.8 μmol/L (range, 1.5–23.0) for blood mercury and blood selenium concentrations, respectively.

We first tested crude correlations between PON1 activity and mercury and selenium concentrations. PON1 activity was positively correlated to both blood selenium (Pearson’s *r* = 0.14; *p* < 0.001) and mercury (*r* = 0.08; *p* = 0.024) concentrations. In addition, PON1 activity was positively correlated with the sum of EPA and DHA content of erythrocyte membranes (*r* = 0.17; *p* < 0.001), HDL-C (*r* = 0.29; *p* < 0.001), LDL-C (*r* = 0.12; *p* < 0.001), and marine mammal fat consumption (*r* = 0.10; *p* = 0.008). In bivariate analyses (Table 2), women displayed higher PON1 activity than did men (*p* < 0.001). The three SNPs of known functionality in the *PON1* gene were all significantly associated with PON1 activity (*p* < 0.001). Alcohol consumption was positively associated with PON1 activity (*p* = 0.046). Participants with diabetes had a lower mean PON1 activity than did those without the disease (*p* = 0.017).

**Table 2:** Geometric mean (GM) PON1 activity (kU/L) according to selected characteristics of participants.

Characteristic	*n*	GM (95% CI) PON1 activity	*p*-Value
Sex						< 0.001
Men		405		10.2 (9.9–10.4)		
Women		491		11.1 (10.8–11.3)		
No. of cigarettes/day						0.531
0		196		10.7 (10.3–11.2)		
1–10		257		10.4 (10.1–10.8)		
11–24		235		10.8 (10.5–11.1)		
≥ 25		100		10.7 (10.2–11.2)		
Frequency of alcohol consumption						0.046
Daily		60		11.5 (10.8–12.2)		
Weekly		157		10.6 (10.3–11.1)		
Monthly		177		10.7 (10.4–11.1)		
Never of yearly		388		10.5 (10.2–10.8)		
Variant and genotype						
rs705379 (–108C/T)						< 0.001
GG (CC)		419		11.4 (11.1–11.6)		
AG (CT)		214		9.8 (9.5–10.1)		
AA (TT)		26		7.5 (6.9–8.2)		
rs662 (Q192R)						< 0.001
GG (QQ)		224		9.9 (9.5–10.2)		
AG (QR)		322		10.9 (10.6–11.2)		
AA (RR)		108		11.8 (11.2–12.4)		
rs854560 (L55M)						< 0.001
TT (MM)		615		10.8 (10.6–11.0)		
AT and AA (LM and LL)		41		9.3 (8.7–10.1)		
Statin intake						0.572
Yes		70		10.4 (9.8–11.1)		
No		826		10.6 (10.4–10.8)		
Cardiovascular disease						0.663
Yes		164		10.5 (10.1–10.9)		
No		732		10.6 (10.4–10.8)		
Diabetes						0.017
Yes		32		9.5 (8.7–10.4)		
No		864		10.6 (10.4–10.8)		
Dyslipidemia						0.694
Yes		71		10.5 (9.8–11.1)		
No		825		10.6 (10.4–10.8)		
CI, confidence interval.						

We used multivariate models to examine the relation between PON1 activity and blood concentrations of mercury and selenium, while taking into account several potential confounders, including *PON1* gene variants previously shown to influence PON1 activity or expression (Table 3). In a model that explained 31.4% of the variance in PON1 activity (*n* = 651), blood selenium concentration remained positively associated with PON1 activity (*p* < 0.001), whereas, in contrast with the bivariate analysis, blood mercury concentration was inversely associated with the activity of the enzyme (*p* < 0.001). Also in contrast with the results of the bivariate analysis, age was inversely associated with PON1 activity in the multivariate model (*p* < 0.001). Sex was not associated with PON1 activity, and we therefore excluded it from the final model. We obtained a similar model for the entire population sample (*n* = 896) after multiple imputation of genotypes (data not shown).

**Table 3:** Multiple linear regression model*a* of plasma PON1 activity (log kU/L) in Inuit adults, Nunavik, 2004.

Variable	β-Coefficient	95% CI	*p*-Value
Age (years)		–0.047		–0.066 to –0.028		< 0.001
Blood mercury (nmol/L)*b*		–0.063		–0.091 to –0.035		< 0.001
Blood selenium (μmol/L)*c*		0.067		0.045 to 0.088		< 0.001
HDL-C (mmol/L)*b*		0.077		0.061 to 0.094		< 0.001
EPA + DHA (%)		0.048		0.023 to 0.074		< 0.001
*PON1* variants						
rs662 (Q192R)						
GG (CC)		–0.189		–0.241 to –0.136		< 0.001
AG (CT)		–0.101		–0.147 to –0.055		< 0.001
rs854560 (L55M)*d*						
TT (MM)		0.096		0.038 to 0.153		0.001
rs705379 (–108C/T)						
GG (QQ)		0.366		0.279 to 0.452		< 0.001
AG (QR)		0.230		0.142 to 0.317		< 0.001
CI, confidence interval. **a**Multiple *R*^2^ = 0.314; *n* = 651. **b**Log-transformed variables. **c**Inverse-transformed variable. **d**Reference category is AT and AA.						

Further adjustment of the multivariate model for blood lead level, fat mass, or traditional food intakes did not modify the relation between blood mercury level and PON1 activity [see Supplemental Material, models 1–3 (doi:10.1289/ehp.1003296)]. We tested the interaction terms between mercury and selenium, mercury and n-3 PUFAs, and mercury and *PON1* gene variants [see Supplemental Material, models 4–6 (doi:10.1289/ehp.1003296)]. None of these interaction terms were statistically significant (*p* > 0.1).

**Figure 2:**
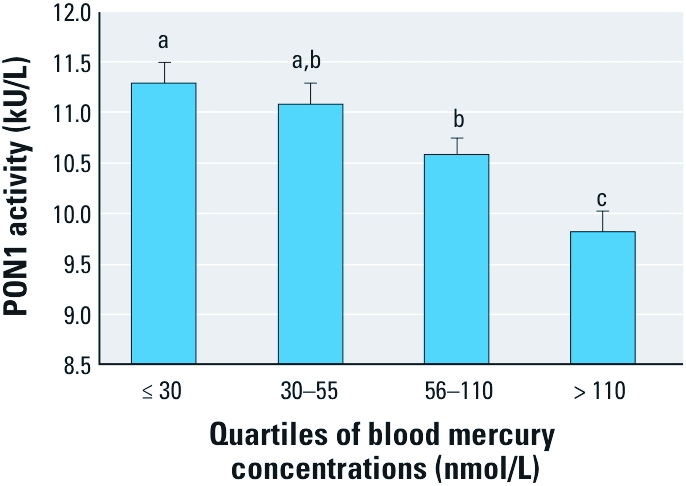
Relation between blood mercury concentrations and PON1 activity in Inuit adults from Nunavik. Each bar represents the geometric mean ± 95% confidence interval. Mean values were adjusted for age, blood selenium levels, plasma HDL-C concentrations, and *PON1* genotypes (rs662, rs854560, rs705379). Different letters above the bars indicate mean values that are significantly different from one another.

Figure 2 presents the relation between blood mercury concentrations (expressed in quartiles) and adjusted geometric mean PON1 activity. Mean activity values decreased in a concentration-dependent fashion with increasing blood mercury concentrations (*p* for trend < 0.001). The geometric mean activity corresponding to the highest blood mercury quartile (> 110 nmol/L; 9.8 kU/L) was 13% lower (*p* < 0.001) than that of the lowest blood mercury quartile (≤ 30 nmol/L; 11.3 kU/L).

## Discussion

In this cross-sectional study, we examined the relationship between blood mercury levels and plasma PON1 activity in a representative sample of the Inuit population of Nunavik, which has been shown to be highly exposed to MeHg mainly through their consumption of marine mammal meat, kidney, and liver (Fontaine et al. 2008). After controlling for several potential confounding factors, we found that increasing blood mercury concentrations were inversely associated with the activity of this enzyme, which protects HDL and LDL from oxidation. Previous evidence for such a relationship had been obtained from *in vitro* studies in which liver microsomes were exposed to various concentrations of the organomercurial compound (Gonzalvo et al. 1997). To our knowledge, this is the first study to report an apparent inhibitory effect of MeHg exposure on PON1 activity in a human population.

Although blood mercury levels showed a weak positive correlation with PON1 activities in bivariate analyses, a multivariate analysis that included several covariates revealed a negative association of blood mercury levels with PON1 activities. This can be explained by the intercorrelations of age, blood selenium concentrations, plasma HDL levels, and the proportion of EPA plus DHA in red blood cell membranes (data not shown), in addition to these variables being linked to blood mercury levels and PON1 activities. Despite these multiple intercorrelations, we avoided collinearity problems in multivariate models by centering and scaling continuous explanatory variables.

We noted that in addition to blood mercury concentrations, age was also inversely related to PON1 activity, as previously reported by others (Cherki et al. 2007; Jarvik et al. 2002; Seres et al. 2004). The negative effect of age on PON1 activity has been attributed to a reduced number of sulfhydryl groups on the protein, because of an increase in oxidative stress in the elderly (Jaouad et al. 2006).

The positive association between HDL-C and PON1 activity was expected because the enzyme circulates in the plasma associated with HDL. This relation has been reported in other studies pertaining to factors that influence the activity of the enzyme in healthy subjects (Boesch-Saadatmandi et al. 2010; Ferré et al. 2003). We also noted a positive influence of EPA plus DHA content of erythrocyte membranes on PON1 activity. The fact that n-3 PUFA intake is significantly linked to PON1 activity in a multivariate model that included HDL-C as a covariate suggests that these fatty acids may increase PON1 activity independently of their known effect on HDL-C levels. Results from an experiment in which rats were fed a diet rich in n-3 PUFAs (13.8% of caloric intake) showed reduced serum PON1 activity compared with rats receiving a diet with a low n-3 PUFA content (2.8% of caloric intake) (Varatharajalu et al. 2010). Although these results suggest that a high intake of n-3 PUFAs can promote oxidation, this may not be the case for a lower intake such as that of the Inuit population.

Three PON1 gene variants that were previously shown to affect the arylesterase activity of the enzyme (Brophy et al. 2001) were also found to influence the activity measured by the hydrolysis rate of the fluorogenic substrate in the present study (Table 3). Based on squared partial correlation coefficients (data not shown), the rs705379 polymorphism had the largest effect on PON1 activity, followed by rs662 and then rs854560. Brophy et al. (2001) reported that the rs854560 effect of lowered arylesterase activity was attributable largely to linkage disequilibrium with the rs705379 polymorphism.

We specifically tested the interaction between blood mercury concentration and the rs662 (PON1_192_) polymorphism because of results from an *in vitro* study indicating that metals, including mercury, might be more effective in inhibiting the PON1_R192_ isozyme (Cole et al. 2002; Costa et al. 2005; Gençer and Arslan 2009). However, we did not find support for such an allele-specific susceptibility to MeHg toxicity, because the mercury × rs662 interaction term was not statistically significant when entered in a multivariate model [see Supplemental Material, model 4 (doi:10.1289/ehp.1003296)].

Results from biochemical studies suggest that the free thiol group on the cysteine-285 residue may be the molecular target of mercurials (Debord et al. 2003; Gonzalvo et al. 1997). Although the cysteine-285 residue does not seem essential for the enzyme activity (Sorenson et al. 1995), binding of MeHg to the thiol group could alter the active site of enzyme and in turn reduce its catalytic activity. This mechanism has been implicated in the inhibition of different enzymes by MeHg, such as thioredoxin (Carvalho et al. 2008) and arylamine *N*-acetyl transferase-1 (Ragunathan et al. 2010). Interestingly, we observed that blood selenium levels appeared to oppose the effect of blood mercury levels on plasma PON1 activity. Selenium is present in the active site of several enzymes, such as thioredoxin reductase, which is involved in redox regulation (Carvalho et al. 2008), and glutathione peroxidase, which protects from oxidative stress (Rayman 2009). Further studies are needed to identify selenium species that are present in selenium-rich food items consumed by the Inuit (e.g., beluga skin) and to decipher the mechanism through which selenium intake might protect against MeHg-induced toxicity in the Inuit population.

A strong point of the present study is its sampling design that allows a proper representation of the Inuit population of Nunavik. Furthermore, to document the precision of the PON1 activity measurement, we included in each analytical batch a plasma sample that had been obtained from a volunteer at the beginning of the study, processed and stored frozen in the same conditions as the population samples. Finally, we determined the genotypes for three SNPs that are known to affect the expression of PON1 or its catalytic activity. This allowed us to verify whether interactions between mercury exposure and *PON1* gene variants that had been noted during *in vitro* experiments could be observed in the Inuit population.

There is no consensus regarding the optimal substrate to measure PON1 activity in relation to cardiovascular diseases. Some authors measured the activity of the enzyme toward paraoxon (Ferré et al. 2003; Mackness et al. 2003), others used phenylacetate (Jayakumari and Thejaseebai 2009), and still others measured the activity toward two substrates, usually paraoxon and diazoxon (Costa et al. 2005). We used a commercial kit, the EnzChek Paraoxonase Assay Kit from Invitrogen, to measure the PON1 activity in plasma samples of our participants. Recently, Yang et al. (2010) reported that PON1 activity, as measured by the same commercial kit, was associated with early structural and functional changes of arteries in a group of 156 hypertensive patients. Furthermore, in the present study, polymorphisms that were previously shown to influence PON1 catalytic activity or expression also modified PON1 activity, in the expected direction. We are looking forward to testing whether PON1 activity as measured by this commercial kit will constitute a useful biomarker to predict the risk of cardiovascular disease in this population during the follow-up phase of the cohort study.

## Conclusion

Results of this cross-sectional study suggest that MeHg exposure exerts an inhibitory effect on plasma PON1 activity. In addition to being a source of exposure to MeHg, marine foods that are part of the traditional Inuit diet are rich in selenium and n-3 PUFAs that may offset inhibitory effects of MeHg on PON1 activity. Dietary changes leading to a decrease in intake of these nutrients could lead to lower PON1 activity and, in turn, increased risk of CHD. Follow-up of the participants is indicated in order to monitor dietary changes and possible related modifications in biomarkers of cardiovascular risk, including PON1 activity.

## Supplemental Material

(16 KB) PDF
