# Supplementary material for: Relation between Methylmercury Exposure and Plasma Paraoxonase Activity in Inuit Adults from Nunavik
Source: Environ Health Perspect. 2011 May 4;119(8):1077–83. doi: 10.1289/ehp.1003296 (PMC3237359; doi:10.1289/ehp.1003296)
Supplement: (16 KB) PDF [file ehp.1003296.s001.pdf]

Supplemental Material

Relation between Methylmercury Exposure and Plasma Paraoxonase Activity in Inuit Adults from Nunavik

Pierre Ayotte, Antoine Carrier, Nathalie Ouellet, Véronique Boiteau, Belkacem Abdous,

Elhadji Anassour Laouan Sidi, Marie-Ludivine Château-Degat, and Éric Dewailly

Supplemental Material, Table 1. Beta coefficients and standards errors for independent variables entered in different multiple linear regression models of plasma PON1 activity (log kU/L) in Inuit adults, Nunavik, 2004

Variables	Model 1 N = 651; R^2 = 0.314[d]	Model 2 N = 599; R^2 = 0.308	Model 3 N = 542; R^2 = 0.328	Model 4 N = 651; R^2 = 0.321	Model 5 N = 651; R^2 = 0.319	Model 6 N = 651; R^2 = 0.318
Age (years)	-0.047 (0.010)***	-0.046 (0.010)***	-0.042 (0.011)***	-0.047 (0.010)***	-0.045 (0.010)***	-0.045 (0.010)***
Blood mercury (nmol/L)[a]	-0.063 (0.014)***	-0.067 (0.015)***	-0.075 (0.014)***	-0.043 (0.055)	-0.068 (0.014)***	-0.070 (0.014)***
Blood selenium (µmol/L)[b]	0.067 (0.011)***	0.062 (0.012)***	0.058 (0.012)***	0.067 (0.011)***	0.071 (0.012)***	0.068 (0.011)***
HDL-C (mmol/L)[a]	0.077 (0.008)***	0.079 (0.009)***	0.073 (0.009)***	0.077 (0.008)***	0.075 (0.008)***	0.076 (0.008)***
EPA+DHA (%)	0.048 (0.013)***	0.040 (0.014)**	0.049 (0.016)**	0.049 (0.013)***	0.049 (0.013)***	0.054 (0.013)***
Blood lead (µmol/L)[a]		0.014 (0.011)				
Fat mass (kg)[a]		0.021 (0.011)*				
Marine mammal fat consumption (g/day)[a]			0.006 (0.009)			
Marine mammal meat consumption (g/day)[a]			0.010 (0.010)			
Country fish consumption (g/day)[a]			0.005 (0.010)			
<u>PON1 variants</u>						
rs662 (Q192R)						
GG	-0.189 (0.027)***	-0.185 (0.029)***	-0.190 (0.030)***	-0.187 (0.027)***	-0.189 (0.027)***	-0.190 (0.027)***
AG	-0.101 (0.023)***	-0.100 (0.024)***	-0.109 (0.025)***	-0.101 (0.023)***	-0.100 (0.023)***	-0.100 (0.024)***
rs854560 (L55M)[c]						
TT	0.096 (0.029)**	0.089 (0.030)**	0.092 (0.031)**	0.109 (0.029)***	0.097 (0.031)**	0.098 (0.029)**
rs705379 (-108C/T)						
GG	0.366 (0.044)***	0.371 (0.043)***	0.390 (0.045)***	0.363 (0.042)***	0.366 (0.044)***	0.367 (0.044)***
AG	0.230 (0.045)***	0.238 (0.045)***	0.248 (0.047)***	0.229 (0.044)***	0.229 (0.045)***	0.229 (0.045)***
<u>Interactions</u>						
PON1 variants x mercury						
rs662 (Q192R)						
GG				-0.006 (0.029)		
AG				-0.040 (0.024)		

rs854560 (L55M)[c]			
TT	0.030 (0.036)		
rs705379 (-108C/T)			
GG	-0.030 (0.043)		
AG	-0.017 (0.042)		
Mercury x selenium		-0.013 (0.008)	
Mercury x EPA+DHA			-0.010 (0.008)

HDL-C: high-density lipoprotein cholesterol; EPA: eicosapentaenoic acid; DHA: docosahexaenoic acid.

[a] Log transformed variables. [b] Inverse-transformed variable. [c] Reference category is AT&AA. [d] Multiple R-square values.

* $p < 0.05$; ** $p < 0.01$; *** $p < 0.001$
